# Proteomic identification of PA2146 as a biofilm marker of *Pseudomonas aeruginosa* on endoscope channel material

**DOI:** 10.1016/j.bioflm.2025.100310

**Published:** 2025-08-06

**Authors:** Koen van der Ploeg, Corné P. de Vogel, Corné H.W. Klaassen, Theo M. Luider, Lona Zeneyedpour, Bibi C.G.C. Mason- Slingerland, Margreet C. Vos, Marco J. Bruno, Michiel L. Bexkens, Juliëtte A. Severin

**Affiliations:** a Dept. of Gastroenterology and Hepatology, Erasmus University Medical Center, Rotterdam, the Netherlands; b Dept. of Medical Microbiology and Infectious Diseases, Erasmus University Medical Center, Rotterdam, the Netherlands; c Clinical and Cancer Proteomics, Dept. of Neurology, Erasmus University Medical Center, Rotterdam, the Netherlands

**Keywords:** Pseudomonas aeruginosa, Biofilms, Proteomics, Mass spectrometry, Matrix-assisted laser desorption-ionization (MALDI-TOF MS), Bacterial proteins, Biomarkers, Duodenoscopes, Endoscopy, Gastrointestinal, Disinfection, Microbial contamination, Instrument contamination

## Abstract

**Study background and aims:**

*Pseudomonas aeruginosa* can persistently contaminate endoscopes by forming biofilms within internal channels, complicating both detection and eradication. Current microbiological surveillance methods have limited efficacy and may yield false-negative results. This study aimed to identify proteomic markers of *P. aeruginosa* biofilms on endoscope channel material.

**Methods:**

Three genetically unrelated *P. aeruginosa* isolates from contaminated duodenoscopes and two reference strains (ATCC 27853 and PAO1) were used. Biofilms were grown on disinfected endoscope biopsy channel rings and incubated for 24, 48, and 72 h. Matrix-assisted laser desorption ionization time-of-flight mass spectrometry (MALDI-TOF MS) was employed to analyze temporal changes in protein spectra. Peaks of interest were further characterized by liquid chromatography–tandem mass spectrometry (LC-MS/MS) and whole-genome sequencing to identify associated proteins. To further confirm the origin of these peaks, strains naturally lacking the corresponding genes were analyzed.

**Results:**

MALDI-TOF MS revealed distinct time- and strain-specific spectral profiles, with two notable peaks at approximately 2723 m/z and 5450 m/z. LC-MS/MS identified the 5450 m/z peak as PA2146, corresponding to a 5449.1 Da protein after in vivo methionine cleavage. The 2723 m/z peak was confirmed as its doubly charged ion. Both peaks were absent in strains naturally lacking PA2146, confirming it as the source.

**Conclusion:**

PA2146 expression increases during *P. aeruginosa* biofilm development on endoscope channel surfaces, indicating its potential as a biomarker for contamination. MALDI-TOF MS could enhance biofilm detection in endoscope surveillance. Further research should assess the clinical utility of proteomic approaches for improving endoscopic microbiological safety.

## 1 Introduction

*Pseudomonas aeruginosa* is a well-known cause of hospital-acquired infections, particularly in immunocompromised patients. Contaminated medical devices, including endoscopes, serve as potential transmission routes, with duodenoscopes implicated in outbreaks involving resistant strains [1]. Contamination rates of up to 30 % have been reported in gastroscopes and colonoscopes [2]. Ready-to-use duodenoscopes show contamination with gut or oral microbiota in 15–19 % of cases [3,4]. Our previous research identified *P. aeruginosa* as one of the most prevalent contaminants in duodenoscopes [5]. *P. aeruginosa* is known for its strong ability to form biofilms that persist in the internal channels of an endoscope. Such biofilms act as robust protective barriers, shielding the bacteria from conventional cleaning agents and leading to persistent contamination despite repeated reprocessing efforts [5,6]. Currently, no effective method exists for removing biofilms from endoscope channels, leaving channel replacement the only viable solution.

To minimize patient exposure to contaminated endoscopes, guidelines recommend routine microbiological surveillance [[7], [8], [9]]. However, current sampling and culturing protocols have demonstrated limited efficacy [10]. This limitation is likely due to biofilm formation in endoscope channels, which impedes the sampling of embedded bacteria and may lead to false-negative culture results [11,12]. This underscores the urgent need for novel detection methods capable of reliably identifying biofilm presence.

*P. aeruginosa* biofilm is structurally complex and dynamic, comprising extracellular DNA, proteins, lipids, and polysaccharides that vary according to strain and environmental conditions [13,14]. Proteomic analyses have revealed that numerous genes and proteins are differentially expressed during biofilm formation, highlighting their roles in biofilm architecture and antimicrobial resistance [15,16]. Among these, a recent study by Kaleta et al. identified PA2146 as a key protein upregulated during biofilm growth in *P. aeruginosa* isolates associated with clinical infections [16].

Identifying proteins and genes uniquely associated with *P. aeruginosa* biofilms in duodenoscope channels may enable the development of more sensitive and targeted detection strategies. Matrix assisted laser desorption ionization-time-of-flight mass spectrometry (MALDI-TOF MS), widely used for bacterial identification, has demonstrated the ability to monitor *P. aeruginosa* biofilm development [17]. Using MALDI-TOF MS, liquid chromatography–tandem mass spectrometry (LC-MS/MS), and whole-genome sequencing analyses, this study aimed to identify proteomic markers of *P*. *aeruginosa* biofilm formation in strains which were found in microbial surveillance cultures of duodenoscopes.

## 2 Materials and methods

### 2.1 Strain selection

Two standard reference strains, ATCC 27853 and PAO1 (designated isolates A and B, respectively), were included due to their widespread use in *P. aeruginosa* research. Both are well-characterized, with publicly available whole genome sequencing (WGS) data enabling comparative analyses [18]. In addition, three genetically distinct clinical isolates (C, D, and E), confirmed by WGS, were selected from a microbiological surveillance database maintained by the Department of Medical Microbiology and Infectious Diseases. Isolates C and E were recovered from separate incidents of persistent contamination in duodenoscope biopsy channels, likely due to biofilm formation. Isolate D, a Verona Integron-encoded Metallo-β-lactamase (VIM)-2 producing strain, was previously linked to a duodenoscope-related outbreak in 2012 [19]. To further support experimental findings, strains F and G, lacking the PA2146 gene, were later included. These were selected from an in-house strain collection based on their genome sequences.

### 2.2 Biofilm generation *in vitro* model

An unused polytetrafluoroethylene (PTFE) biopsy channel from a Pentax ED34-i10T2 duodenoscope was sectioned into 4 mm rings. A biopsy channel was used because it is one of the most frequently contaminated duodenoscope sites [5,20]. The 4 mm size was chosen for compatibility with 48-well plates, facilitating efficient experimental handling. To ensure uniformity, a custom PTFE tube slicer was designed and 3D-printed specifically for this purpose (Supplementary appendix Figure S1). Biopsy channel rings (BCRs) were disinfected by immersion in 70 % ethanol for 24 h, then air-dried in a sterile flow cabinet. Fresh overnight cultures of all isolates were grown on Tryptic Soy Agar (TSA; Becton, Dickinson and Company, Franklin Lakes, NJ, USA) and incubated at 37 °C for 24 h. Disinfected BCRs were placed horizontally in 500 μL of Tryptic Soy Broth (TSB; same supplier), and inoculated with 10 μL of a 0.5 McFarland suspension prepared from a single-strain overnight culture. Incubation was carried out for 24, 48, and 72 h. To enable simultaneous termination, the 72-h condition was initiated on day 0, the 48-h on day 1, and the 24-h on day 2. On day 3, all BCRs were removed and thoroughly washed in 0.9 % NaCl to eliminate loosely attached material.

### 2.3 Mass spectrometry

**Fig. 1:**
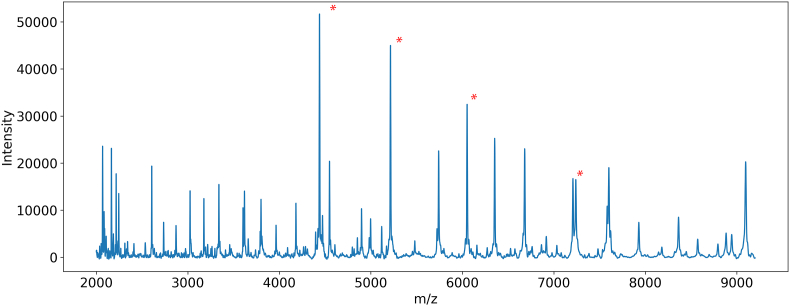
MALDI-TOF mass spectrum of *Pseudomonas aeruginosa* from blood agar plate (ATCC 27853). Unique molecular fingerprint of *Pseudomonas aeruginosa* obtained by MALDI-TOF MS. Each peak represents a specific molecular ion, primarily ribosomal proteins and other abundant biomolecules. The x-axis (*m/z*) indicates the mass-to-charge ratio, while the y-axis shows relative intensity, reflecting ion abundance. Detected masses correspond to proteins encoded by the bacterial genome, providing insights into molecular composition and structure. ∗ mark known ribosomal proteins, which serve as molecular markers for species identification and as internal references to detect relative changes in other protein abundances.

For MALDI-TOF (Bruker, Bremen, Germany) analysis, protein extraction was performed using formic acid and acetonitrile [21]. Briefly, BCRs were washed and transferred to Eppendorf tubes containing 300 μL of water, followed by the addition of 900 μL of water. After centrifugation at 22,000g for 5 min, the supernatant was discarded, and BCRs were briefly dried under a nitrogen stream. Subsequently, 20 μL of 70 % formic acid was added, and BCRs were incubated for 5 min, after which 20 μL of acetonitrile was added. Following a second centrifugation at 22,000g for 5 min, 1 μL of the supernatant was spotted in triplicate onto a MALDI Biotarget (Bruker, Bremen, Germany), air-dried, and overlaid with 1 μL of α-cyano-4-hydroxycinnamic acid (CHCA) matrix solution. Once dried, the target plate was loaded into the MALDI-TOF. Spectra were recorded in linear mode within a mass-to-charge (*m/z*) range of 2K–20K, at a laser intensity of 35 %, using a recording summing 200 shots, to a total of 800 shots per spectrum. This range is widely used for detecting peptides and proteins with masses between 2000 Da and 20,000 Da, including multiple ribosomal proteins (Fig. 1). This approach enables precise genus and species identification and facilitates the detection of biofilm-associated proteins. All reagents were of analytical grade and purchased from Sigma-Aldrich, unless stated otherwise.

For LC-MS/MS analysis, the incubation and processing of BCRs were performed identically to those analyzed with MALDI-TOF MS, except that protein extraction was replaced by trypsin treatment. Following a water/ethanol wash, the supernatant was discarded, and BCRs were treated with 50 μL of 5 % sodium deoxycholate (SDC) solution (Sigma-Aldrich, St. Louis, MO, USA). Samples were subjected to ultrasonic bathing (42 kHz) for 5 min at room temperature, followed by incubation at 80 °C for 10 min with shaking at 450 rounds per minute (rpm) in an Eppendorf ThermoMixer C (Eppendorf, Hamburg, Germany).

Subsequently, 450 μL of MS-grade water (Fisher Chemical, Waltham, MA, USA) was added to each sample. Trypsin (5 μL of 1 μg/μL; Trypsin Gold, Worthington Biochemical Corporation, NJ, USA) was then added, and the tubes were incubated at 37 °C for 1 h with shaking at 450 rpm. Following incubation, 12.5 μL of 5 % trifluoroacetic acid (TFA; Thermo Fisher Scientific, Waltham, MA, USA) was added to each tube, resulting in immediate protein precipitation. Tubes were briefly vortexed and centrifuged at maximum g-force for 5 min using a 5424 refrigerated centrifuge (Eppendorf, Hamburg, Germany).

Mass spectrometry measurements were performed using a capillary liquid chromatography system (Thermo Fisher Scientific, Germany) online coupled to an Orbitrap Fusion Lumos Mass Spectrometer (Thermo Fisher Scientific, San Jose, CA, USA), as described previously [22]. From the raw data files, MS/MS spectra were converted into mascot generic format (MGF) files using ProteoWizard (version 3.0.22130). MGF peak lists were searched against the uniprot_sprot_v201212 database (restricted to bacteria; 334,772 sequences) using Mascot (version 2.3.01; Matrix Science, London, UK). Mascot search results were further analyzed in Scaffold (version 5.3.2, Portland, OR, USA), with protein and peptide confidence levels set to a 1 % false discovery rate (FDR). FDRs were estimated by including a decoy database search generated by Mascot.

### 2.4 Whole genome sequencing

To support the mass-spectrometry analysis, and to confirm gene/protein relation, WGS of all strains involved was performed. Genomic DNA was isolated from freshly grown cultures of the study isolates using the Quick-DNA Fungal/Bacterial Miniprep Kit (Zymo Research via Baseclear, Leiden, Netherlands). Libraries of ∼350 base pairs fragments were created and sequenced by Novogene (Cambridge, United Kingdom) on Illumina platforms (Illumina, San Diego, CA, USA) generating 150 base pairs paired end reads. *De novo* assemblies were created using CLC Genomics Workbench v21 (Qiagen, Hilden, Germany) using default parameters. To examine presence of and variation within the target sequence and to overcome possible assembly issues based on short read sequencing alone (e.g. false negatives), both an assembly-based and an assembly-free protocol were used. In the assembly-based protocol, assemblies were imported into BioNumerics v7.6 software. The sequence extraction module was used to extract the target sequence from the assemblies based on a minimal length coverage of 95 % and minimal sequence identity of 80 % to the reference sequence. Extracted sequences were translated and aligned to identify amino acid variants. In the assembly-free protocol, sequence reads were mapped onto the reference sequence in CLC Genomics Workbench using default parameters. From these mappings, a consensus sequence was generated, translated and aligned to identify amino acid variants.

### 2.5 Protein identification

To identify MALDI-TOF MS peaks associated with biofilm development, spectra from isolates A to E were acquired at the three time points. Peaks were evaluated for temporal intensity changes relative to ribosomal protein peaks, and those consistently increasing across all isolates were selected for further analysis. To determine the proteins corresponding to these peaks, a search was performed in the UniProt-reviewed Swiss-Prot proteome database (taxonomy ID: 208964) to match detected masses with annotated proteins. In parallel, LC-MS/MS analysis was conducted, and the resulting spectra were compared against a database of known protein and peptide masses. Candidate proteins were cross-referenced with WGS data to confirm their presence in the isolates. Finally, isolates F and G, naturally deficient in the PA2146 gene, were analyzed by repeating the MALDI-TOF MS biofilm protocol to assess the impact of gene deletion on the spectral profile.

## 3 Results

**Fig. 2:**
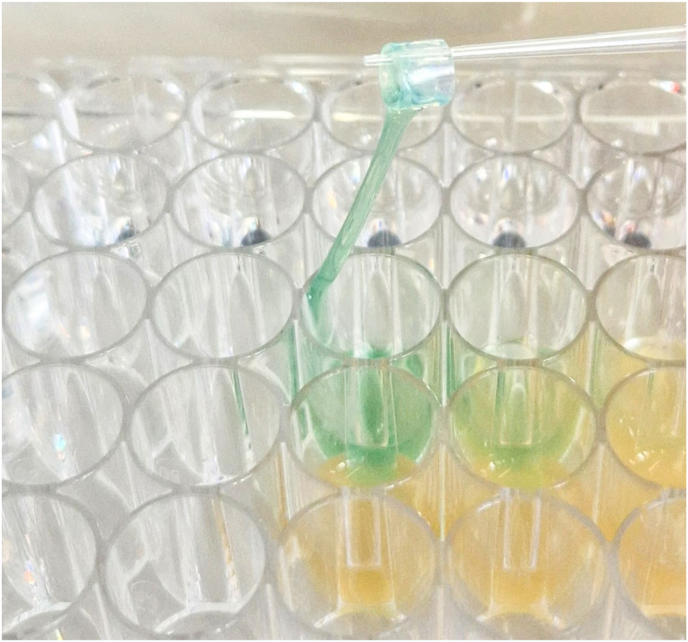
*Pseudomonas aeruginosa* biofilm formation on a biopsy channel ring (BCR). The pipette tip holds a BCR from the 72-h time point.

*P. aeruginosa* reference strains A and B, along with clinical isolates C, D, and E, were incubated on BCRs for 24, 48, and 72 h. Biofilm formation was observed in all samples. Visual inspection confirmed extensive biofilm development, with the medium transforming into a dense, slime-like matrix (Fig. 2). This was accompanied by blue, green, or brown discoloration, attributed to the production of pyoverdine and pyocyanin, secondary metabolites commonly associated with *P. aeruginosa* biofilm development [13].

### 3.1 Temporal and strain-specific variations in MALDI-TOF MS spectra

**Fig. 3:**
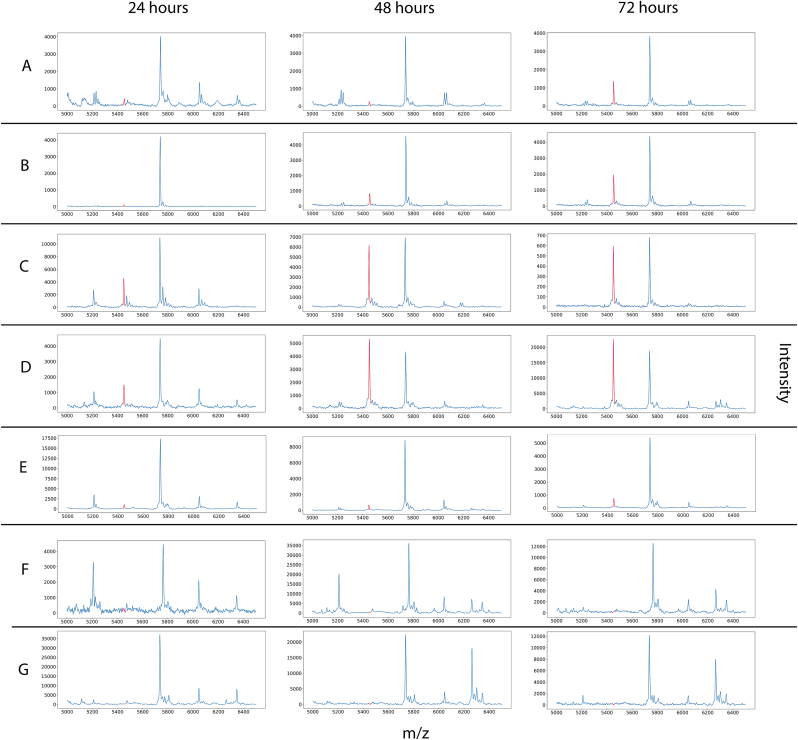
MALDI-TOF mass spectra of six *Pseudomonas aeruginosa* strains recorded at 24, 48, and 72 h post-incubation. The 5450 m/z peak (highlighted in red) is shown relative to 5739 m/z, to which the y-axis is scaled, unless the 5450 m/z had a higher intensity (Isolate D). Rows represent individual strains, and columns correspond to time points. The x-axis denotes mass-to-charge ratio (*m/z*), and the y-axis represents signal intensity of the individual graphs.

MALDI-TOF MS spectra were generated for all strains across the three time incubation points (24, 48, and 72 h), and compared both over time, and between samples. Distinct differences in spectral patterns were observed between strains and over time. Two peaks of particular interest, at 2723 m/z and 5450 m/z, showed clear temporal and strain-specific trends. In control strains A and B, these peaks were of low intensity at 24 h but increased significantly by 48 and 72 h. In clinical isolates, particularly isolate D, these peaks were already present at high intensities at 24 h, further intensifying over time (Fig. 3). The consistent spectral trend across replicates underscores their biological relevance.

### 3.2 Protein identification

**Fig. 4:**
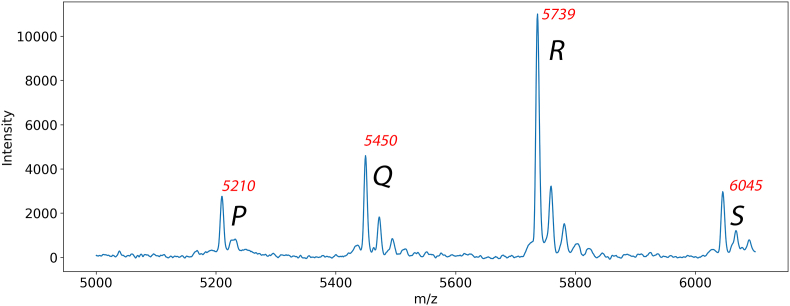
Representative MALDI-TOF MS spectrum of *Pseudomonas aeruginosa* strain B, highlighting the 5000–6100 m/z region. Peaks P (5210 m/z), Q (5450 m/z), R (5739 m/z), and S (6045 m/z) correspond to proteins listed in Table 1, with Peak Q (5450 m/z) identified as the biofilm-associated protein PA2146.

Using WGS data, we attempted to identify the 5450 m/z peak. However, no direct candidates were found, as no genes encoding a protein with a final mass of 5450 Da were present in the sequenced genomes. By comparing MALDI-TOF MS spectra with LC-MS/MS analysis, we identified four proteins within the 5000–6100 Da region (Table 1). A representative MALDI-TOF MS spectrum is shown in Fig. 4. Three of the four detected proteins, bL34, bL33, and bL7, could be directly correlated with a MALDI-TOF MS peak. For the fourth protein, PA2146, no direct 1:1 correlation was initially observed based on its mass. However, further analysis of the LC-MS/MS data revealed that PA2146 had undergone in vivo methionine cleavage, a common post-translational modification in bacterial proteins [23]. This modification reduced the actual mass of the in vivo protein to 5449.1 Da, aligning with the detected peak at 5450 m/z, thereby confirming PA2146 as the corresponding protein (Table 1).

**Table 1:** Proteins identified within the 5000–6100 Da region of MALDI-TOF MS spectra were based on consistent detection across isolates. Peaks were further analyzed via LC-MS/MS, and proteins were marked as detected when corresponding peptides were identified.

Measured peak + predicted mass (Da)	Protein name	MALDI-TOF MS	LC-MS/MS
A. 5210	Large ribosomal subunit protein bL34	✓	✓
B. 5580/5450^a^	Biofilm-associated protein PA2146	✓	✓
C. 5739	Ribosomal protein bL7	✓	✗
D. 6045	Large ribosomal subunit protein bL33	✓	✓

✓ indicates successful detection ✗ denotes no detection.

a Represents the mass of PA2146 after in vivo methionine cleavage.

Additionally, the peak at approximately 2723 m/z was identified as the doubly charged ion of PA2146, calculated based on the molecular weight of its singly charged species at 5450 m/z and accounting for ionization mass [24].

### 3.3 Confirmation of PA2146

To further confirm the identity of peak Q as PA2146, we searched our in-house database of sequenced *P. aeruginosa* genomes for strains lacking the PA2146 gene. Two such strains were retrieved from storage, cultured on agar, and analyzed. As they do not encode PA2146, these strains are incapable of producing the corresponding protein. Repetition of the time-series experiment with the strains lacking PA2146 (strains F and G) confirmed the specificity of this peak, as neither 5450 m/z nor its doubly charged ion at 2723 m/z was detected under identical conditions at any time-point (Fig. 3). Strains F and G were still able to form biofilm, indicating that absence of PA2146 does not entirely impair biofilm formation.

## 4 Discussion

This study identifies PA2146 as a biofilm-associated protein upregulated during *P. aeruginosa* biofilm maturation on PTFE duodenoscope biopsy channel rings. Detection was performed using MALDI-TOF MS, a widely accessible and cost-effective proteomic platform. To our knowledge, this is the first application of proteomic biofilm profiling in the context of endoscope contamination. These findings suggest the potential of PA2146 as a biomarker for biofilm detection and underscore the need to clarify its functional role in biofilm development, which may support targeted strategies to prevent or disrupt biofilms in endoscopic devices.

Multiple studies have illustrated the upregulation of PA2146 in biofilm development of *P. aeruginosa* clinical strains [14,16]. A recent study identified PA2146 as the most consistent and substantial upregulated gene during *P. aeruginosa* biofilm development [16]. The same study also illustrated that PA2146 is an important gene involved in biofilm architecture and the resistance of *P. aeruginosa* in biofilm to tobramycin and hydrogen peroxide. Homologs of PA2146 gene have also been discovered in other Proteobacteria, such as *Klebsiella pneumoniae* and *Escherichia coli*, where it similarly influences biofilm formation and antibiotic tolerance. However, the precise role of the PA2146 protein in extracellular matrix production, cell adhesion, or intercellular communication within biofilms remains unclear. Our findings show that PA2146 is also consistently upregulated during biofilm formation on endoscope biopsy channel material in isolates cultured from contaminated endoscopes. Nevertheless, *P. aeruginosa* isolates lacking PA2146 are still capable of forming biofilm. Therefore, sample collection for culture remains essential for detecting endoscope contamination, even if detection of PA2146 through MALDI-TOF MS were to be implemented in clinical practice. Furthermore, future research should investigate the expression and functional role of PA2146 homologs in other common endoscope-associated pathogens to assess their potential as cross-species biofilm biomarkers.

Currently, the detection of endoscope contamination relies on endoscope sampling and culturing [9]. However, the sensitivity of these cultures is limited, with false-negative results occurring in up to 17.8 % of cases [12,25]. *P. aeruginosa* is detected in up to 7.2 % of surveillance cultures from duodenoscopes and has been implicated in multiple duodenoscope-associated outbreaks worldwide [5,19,26,27]. Biofilm formation is believed to play a major role in persistent duodenoscope contamination, particularly in the case of build-up biofilms, which are formed as concentric layers over successive reprocessing cycles [6]. In such a build-up biofilm, *P. aeruginosa* can remain embedded in a viable but nonculturable (VBNC) state for up to 26 weeks of dry storage [11]. Consequently, there is an urgent need for the development and implementation of a biofilm detection method to enhance surveillance efforts and facilitate the early identification of persistent contamination. In future experiments, the BCR model developed in this study could be modified to more closely mimic build-up biofilm accumulation. This could be achieved by incorporating simulated reprocessing steps used for clinically employed endoscopes, including brushing, flushing, and rinsing with detergents and disinfectants such as peracetic acid. This will allow a further assessment of changes in the proteomic profile including the presence of the PA2146 peak.

MALDI-TOF MS offers high specificity and sensitivity for *P. aeruginosa* detection and identification even in the presence of genetic mutations [28]. Furthermore, it has previously been demonstrated to effectively differentiate between various stages of biofilm development, providing valuable insights into the dynamics of biofilm maturation and persistence [17]. Its relatively low cost and broad availability make MALDI-TOF MS a practical and accessible tool with potential for routine clinical applications, including microbiological surveillance of endoscopes. Notably, the analysis can be completed in under 1 h. However, to enable the practical use of MALDI-TOF MS in surveillance, extraction methods tailored for intact, clinically used endoscopes should be developed. Future studies should assess whether MALDI-TOF MS can be used to reliably detect PA2146 in samples collected from endoscopes persistently contaminated with *P. aeruginosa* using the flush-brush-flush method, a standard technique for duodenoscope sampling [10]. Moreover, the methods used in this study may help to identify potential biomarkers in other gastrointestinal microbiota, broadening the applicability of our approach to other biofilm-forming organisms.

There are several limitations to this study. This was a single center study using a limited number of clinical isolates which may impact generalizability. Additionally, the rings of the biopsy channels in our experiment were not subjected to endoscope reprocessing protocols and thus were not exposed to the detergents and chemicals that might influence biofilm formation [29]. Furthermore, in order to maximally stimulate biofilm formation, the biofilms were grown with ample nutrients, which does not accurately reflect the nutrient-poor environment of a biopsy channel from a fully reprocessed and dried endoscope. As a result, our *in vitro* model may differ from biofilms typically present in endoscopes, where repeated reprocessing cycles can lead to the development of build-up biofilms with distinct structural properties. The upregulation of proteins specifically associated with such biofilms may have been missed. This study focused on biofilms in the biopsy or suction channels of endoscopes, where a flush-brush-flush method can potentially be used to assess PA2146 expression. However, due to the significantly narrower diameter of air/water channels, the flush-brush-flush method is not feasible. Therefore, alternative sampling techniques will need to be developed to evaluate PA2146 upregulation in these channels. While PA2146 was significantly upregulated during biofilm formation in clinical isolates, its expression may also be influenced by environmental factors or microbial competition within the endoscope. Stress conditions, such as nutrient limitation, oxidative stress, or exposure to antimicrobial agents, could similarly induce PA2146 expression, although this was not observed in a previous study [16]. Further research is needed to assess whether our experimental conditions contributed to the observed trends and to determine how well they replicate the environment of reprocessed and dried duodenoscope channels.

## 5 Conclusion

The upregulation of PA2146 indicates *P. aeruginosa* biofilm maturation on endoscope biopsy channel material and may serve as a biomarker for detecting biofilm-associated contamination in endoscopes. Future studies are needed to determine whether targeted detection of PA2146 is feasible in clinical samples from endoscope channels and whether its presence correlates with viable *P. aeruginosa* isolates.

## CRediT authorship contribution statement

**Koen van der Ploeg:** Writing – review & editing, Writing – original draft, Validation, Project administration, Investigation, Conceptualization. **Corné P. de Vogel:** Writing – review & editing, Validation, Resources, Investigation. **Corné H.W. Klaassen:** Writing – review & editing, Validation, Methodology. **Theo M. Luider:** Writing – review & editing, Resources, Investigation. **Lona Zeneyedpour:** Investigation. **Bibi C.G.C. Mason- Slingerland:** Writing – review & editing, Supervision. **Margreet C. Vos:** Writing – review & editing, Supervision. **Marco J. Bruno:** Writing – review & editing, Supervision. **Michiel L. Bexkens:** Writing – review & editing, Writing – original draft, Visualization, Validation, Supervision, Software, Methodology, Investigation, Formal analysis, Data curation, Conceptualization. **Juliëtte A. Severin:** Writing – review & editing, Supervision, Methodology, Conceptualization.

## Declaration of competing interest

The authors declare the following financial interests/personal relationships which may be considered as potential competing interests: Margreet C. Vos reports a relationship with Boston Scientific Corporation that includes: funding grants. Marco J. Bruno reports a relationship with Boston Scientific Corporation that includes: consulting or advisory, funding grants, and speaking and lecture fees. Marco J. Bruno reports a relationship with Cook Medical Inc that includes: consulting or advisory, funding grants, and speaking and lecture fees. Marco J. Bruno reports a relationship with PENTAX Medical that includes: consulting or advisory, funding grants, and speaking and lecture fees. Marco J. Bruno reports a relationship with Mylan Pharmaceuticals Inc that includes: funding grants. Marco J. Bruno reports a relationship with ChiRoStim that includes: funding grants. Marco J. Bruno reports a relationship with Ambu that includes: funding grants. If there are other authors, they declare that they have no known competing financial interests or personal relationships that could have appeared to influence the work reported in this paper.

## Data Availability

Data will be made available on request.

## Appendix A Supplementary data

Fig. SS1 A 3D-printed polytetrafluoroethylene tube slicer, used with a sterile scalpel, to ensure precise sectioning Fig. SS1
