# Supplementary material for: Proteomic identification of PA2146 as a biofilm marker of Pseudomonas aeruginosa on endoscope channel material
Source: Biofilm. 2025 Aug 6;10:100310. doi: 10.1016/j.bioflm.2025.100310 (PMC12355587; doi:10.1016/j.bioflm.2025.100310)

**Supplementary appendix**

**Figure S1** A 3D-printed polytetrafluoroethylene tube slicer, used with a sterile scalpel, to ensure precise sectioning


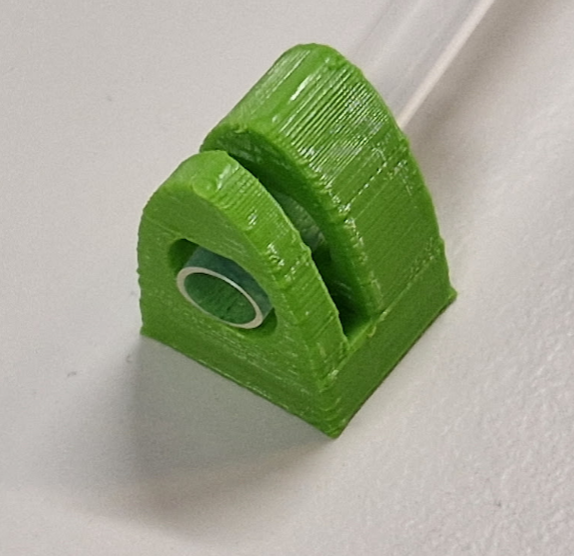

Supplement: Fig. SS1 — A 3D-printed polytetrafluoroethylene tube slicer, used with a sterile scalpel, to ensure precise sectioning [file mmc1.docx]
